# Supplementary material for: Validation of conventional PCR-like alternative to SARS-CoV-2 detection with target nucleocapsid protein gene in naso-oropharyngeal samples
Source: PLoS One. 2021 Sep 23;16(9):e0257350. doi: 10.1371/journal.pone.0257350 (PMC8459981; doi:10.1371/journal.pone.0257350)
Supplement: Attachment — Submitted filename: Response to Reviewers.docx [file pone.0257350.s002.docx]

Response to Reviewers

PONE-D-21-11819

Validation of conventional PCR-like alternative to SARS-CoV-2 detection with target nucleocapsid protein gene in naso-oropharyngeal samples

Editor Comments:

Dear Dr. Jean-Luc Darlix,

Thank you for your availability and attention in editing our manuscript.

We fully accept the suggestions and respond to all, from the editor and reviewer.

We also include as an author, in this review version, Prof. Fabrício Campos, PhD, a virologist who also contributed to the study.

We do not find it necessary to deposit our protocol in the recommended domain ([protocols.io](http://protocols.io/)) since our methodology is very descriptive and allows for full reproducibility of all analyzes performed.

A peer-reviewed Lab Protocol article submission we also think it would frame as self-plagiarism.

To avoid any problems in writing in English, we forward the file to a company specialized in academic review. The revision certificate is attached.

We update epidemiological data from Brazil in the introduction.

Specific comments are identified by the authors (AU):

*Specifically, please summarize the NC functions and the immune responses in natural infections. Relevant references on the viral nucleocapsid N protein are from Luis Enjuanes et al. showing that N is a multifunctional protein with RNA binding and chaperoning activities. In addition specific IGG against N are found in people who experienced Cov infection.*

AU: We have fully included your suggested and reference in the introduction, Line 55 to 55, highlighted in yellow.

Reviewer 1:

*This is an interesting paper that describes an alternative, PCR-based method for COVID-19 diagnosis, beyond the predominant real-time PCR methods that are being utilized worldwide. While the total amount of data presented and overall scope of the work is limited, the main point of the paper is important and timely. Thus, while this manuscript is much shorter than most full-length articles, I recommend publishing, due to the importance of the findings.*

AU: Dear reviewer, thank you for your evaluation.

We are happy for the recognition of the merit of our laborious study, executed under many difficulties in north of Brazil in the current situation.

Our goal has always been the most objective and accurate wording possible. This justifies our shorter format, making it easier to read and interpret the result for the reader.

All authors are grateful for your valuable and important collaboration in evaluation of the manuscript.
